# An alternative approach to kinetic analysis of temperature-programmed reaction data†

**DOI:** 10.1039/c7ra09848k

**Published:** 2018-01-16

**Authors:** A. S. Portnyagin, A. P. Golikov, V. A. Drozd, V. A. Avramenko

**Affiliations:** Department of Sorption Processes, Institute of Chemistry, Far Eastern Branch of Russian Academy of Sciences pr. 100-letiya Vladivostoka Vladivostok Russia arsuha@gmail.com; School of Natural Sciences, Far Eastern Federal University Sukhanova str 8. Vladivostok Russia; Scientific Educational Center of Nanotechnology, Far Eastern Federal University Sukhanova str 8. Vladivostok Russia

## Abstract

To date, kinetic computations have been carried out efficiently for a great variety of physico-chemical processes including crystallization, melting and solid–solid transitions. However, appropriate methods for the kinetic analysis of chemical reactions, especially multi-staged reactions, are currently lacking. Here we report on an alternative way of treating temperature-programmed reaction data using the reduction of iron(iii) oxide as an example. The main principle in the suggested approach is to take into account every stage of the studied process, resulting in a system of kinetic differential equations. Kinetic parameters (activation energy and preexponential factors) are optimized for each of the stages, and cubic splines are used to approximate the conversion functions that reflect changes in reaction-specific surface area throughout the process. The applicability of the suggested method has been tested on temperature-programmed reduction (TPR) data for iron(iii) oxide samples produced from the original Fe_2_O_3_ powder by annealing it at 600, 700 and 800 °C. Results of kinetic analysis obtained at different temperature regimes demonstrate the good stability and performance of the method. Peculiarities of iron(iii) oxide reduction have been revealed, depending on the stage and heating rate. The influence of material morphology on the reduction kinetics has been assessed by comparing preexponential factors corresponding to the first reduction stage. This approach allows a comparison of the structural characteristics of the materials based on the kinetic analysis of the TPR data. Using optimized conversion functions, the initial particle size distribution has been reproduced. Theoretically found particle size distribution was found to correlate well with the experimental distribution obtained *via* laser diffraction.

## Abstract 2

Presented method of kinetic analysis of non-isothermal reaction data provides precise kinetic parameters for different materials with different morphology and particle size.

## Graphical abstract

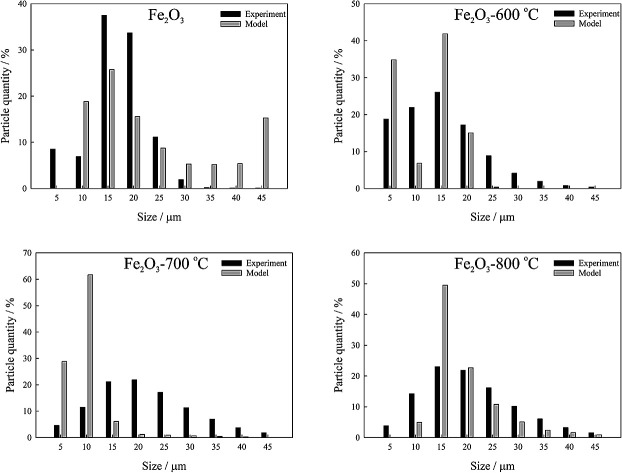

## 1 Introduction

Experimental techniques for thermal analysis are widely used in the characterization of solids, enabling one to trace processes occurring during the thermal treatment of the material. Investigations of thermic decomposition, phase transformations and heterogeneous chemical reactions are conducted most effectively by non-isothermal methods.^1–3^ Information about the dynamics of such processes allows the targeted selection of conditions for the fabrication of more effective functional materials. Methods of temperature-programmed reactions are convenient instruments in a researcher's toolbox for collecting such information.^4–6^ The main advantages of this group of methods include the simplicity and accuracy of experimental apparatus and procedure: the gas mixture, consisting of reactive and inert gases, flows through the investigated sample under heating at a constant rate. The concentration of the reactive gas is measured during the experiment at the outlet. Most frequently used variations of this group of methods are temperature-programmed reduction and oxidation (TPR and TPO), deriving from the importance of the reduction or oxidation step in the catalyst production cycle. A remarkable feature of the TPR method is the possibility of comparing the dispersion of oxides prepared by different methods.^7^ In some cases, it is possible to determine the quantity of the oxide phase in the investigated sample.^8^ According to the location of the peak maximum in the TPR curve, one can compare different samples, revealing strong metal–support interactions and the influence of the support on the catalyst operation.^9–12^

Despite the wide scope of TPR applications in investigational practice, analysis of experimental results is carried out far more superficially. As usual, conclusions are based on such facile assessments as analysis of peak shape alterations or changes in the location of the peak maxima of the TPR curve.^13–15^ However, more detailed information about the underlying processes of temperature-programmed reactions can be obtained by a quantitative description of the TPR processes conducted *via* kinetic analysis.^16^

There are two main groups of method used to process non-isothermal experimental data: isoconversional and model fitting methods. These methods are commonly based on eqn (1):1
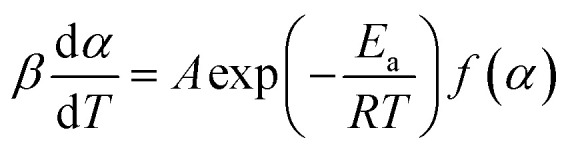
where *α* is the conversion degree, *β* is the heating rate (kelvin per second), *T* is the absolute temperature in kelvin that changes *via T* = *T*_0_ + *βτ*, *τ* is time in seconds; *A* is a preexponential factor (s^−1^), *E*_a_ is activation energy and *R* is the gas constant. Thus, the temperature function of the rate constant is described by Arrhenius law and the conversion function, *f*(*α*), which is frequently associated with the mechanism of heterogeneous reaction or kinetic model, defining the dependence of the reaction rate on the degree of conversion.

The basic assumption of isoconversional (model-free) methods is that the reaction rate depends only on temperature at the same degree of conversion. In order to implement this assumption, it is necessary to have experimental data recorded at several heating rates. Processing non-isothermal experimental data is carried out by means of various model-free methods.^17^ In the method suggested by Friedman,^18^ the logarithmic representation of rate eqn (1) is used:2
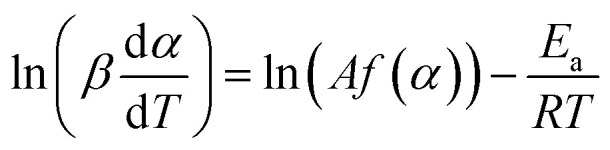


Then, for a chosen *α*, the plot ln(*β*d*α*/d*T*) *vs. T*^−1^ obtained from the TPR curves recorded at different heating rates is built up. If it is a straight line, its slope allows evaluation of the activation energy.

The Flynn–Wall–Osawa method,^19^ based on the integral form of eqn (1), includes Doyle's approximation for temperature integral:^20^3
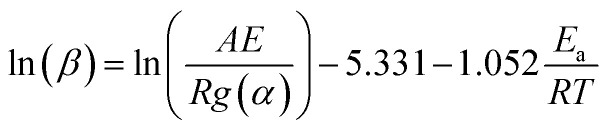


The procedure for evaluating activation energies is similar to the procedure for the Friedman method.

If the process is characterized by one rate-limiting step or several separated in time, then it is possible to obtain a unique set of kinetic parameters. However, in the case of a blend of oxides, in which reduction is not a separate process, or oxide reacting through multiple intermediate stages (*e.g.* reduction of iron oxide), kinetic analysis performed by isoconversional methods gives us variable (apparent) *E*_a_, which depends on *α*. Although it is useful to use isoconversional kinetics to reveal the process mechanism, evaluation of the apparent activation energy and conversion function is complicated due to the absence of sound theory behind chemical solid–gas reactions. Even though there are a lot of mathematical expressions for *f*(*α*), each of which refers to a certain type of topochemical mechanism, it is difficult to choose between them due to the fact there can be several mechanisms acting when the wide temperature region is studied. Also, several kinetic models can fit well and be equally accurate but correspond to different activation energies and preexponential factors. Further, in the case of the multistage process mentioned above, each stage can be characterized by an individual kinetic triplet (*E*_a_, *A*, *f*(*α*)), which cannot be addressed by eqn (1).

In the case of a multistage process, it is advisable to implement methods of model fitting which imply a numerical solution to the system of differential equations, each of which corresponds to one of the stages.^21^ Thus, the TPR curve is simulated while the kinetic parameters are adjusted to fit the experimental curve. However, because of the correlation between kinetic parameters, results of model fitting strongly depend on the initial approximation.^22,23^ Consequently, if no additional assumptions about the process are made, it is impossible to achieve the unique set of kinetic parameters *via* model fitting methods.

Here we present an attempt to overcome the aforementioned disadvantages of existing methods of kinetic analysis. The aim of this study was to create a universal approach for the kinetic analysis of non-isothermal solid–gas reaction experimental data, based on minimal model assumptions. The key point of the suggested method is to reproduce functions of “active” specific surface area *S*(*α*) (where *α* is conversion degree) from the set of TPR experimental curves. The suggested method allows separating temperature (Arrhenius) dependence and temperature independent (specific surface area changes) parts of the TPR curve and, therefore, obtaining the possibility of determining stable (mathematically) activation energies of separate stages in a multistage TPR process. TPR experimental data of iron(iii) oxide were selected to test the suggested method.

## 2 Experimental

### 2.1 Materials and methods

Iron(iii) oxide (99.98%) powder was purchased from Sigma-Aldrich. To prove the influence of sample morphology on the results of the kinetic analysis, a comparison of several samples with different morphologies and surface areas was conducted. Thus, the original Fe_2_O_3_ sample was annealed at 600, 700 and 800 °C for 3 hours.

The Brunauer–Emmett–Teller (BET) surface area of the iron oxides was determined on a Autosorb IQ (Quantachrome, USA) device. Scanning electron microscope (SEM) images were taken using a Carl Zeiss Crossbeam 1540xb (Germany) instrument. Particle size distributions were obtained using Fritsch particle sizer Analysette 22 (Germany). TPR measurements were carried out on an automated chemisorption analyzer ChemBET Pulsar TPR/TPD (Quantachrome Instr., USA). Sample powders (35–40 mg) were loaded into the quartz sample cell. To separate water forming during oxide reduction, a liquid nitrogen cold trap was used. Prior to the experiment, samples were annealed at 350 °C in a nitrogen flow for 30 minutes to degas and remove moisture. TPR curves were recorded under different temperature programs, at a flow rate of 50 mL min^−1^, under flow of 6% H_2_ + N_2_ and ambient pressure. Purity of gases was 99.995 vol%.

## 3 Theory

### 3.1 Model description

The main disadvantages of existing methods of non-isothermal kinetic analysis include low versatility with respect to complex systems, whose reaction kinetics is not fully described by eqn (1) and low reproducibility due to the strong influence of the initial approximation on the final results. In the present work, we suggest a universal approach to the kinetic analysis of heterogeneous reaction data, obtained in non-isothermal conditions. The main principle is based on the reproduction of relative specific surface functions *S*(*α*) from the set of experimental TPR curves. Obtained kinetic parameters are used to characterize investigated materials.

In TPR experiments, small amounts of solid material and high gas flow rates are usually used. In connection to this, the following assumptions are made: (1) because reaction by-products (in the case of TPR–water) are removed from the reaction zone with gas flow, reverse reaction can be neglected; (2) curve broadening caused by longitudinal diffusion is not to be taken into account; (3) the elementary stage of the reduction reaction is first order with respect to hydrogen.

Let's consider the kinetic model of the reduction of bivalent metal with hydrogen that corresponds to the scheme MeO + H_2_ = Me + H_2_O. Hydrogen consumption over time, d*τ*, is defined by eqn (4):4
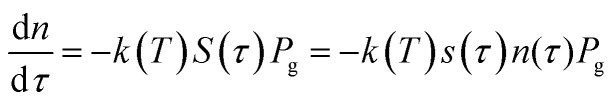
where *k*(*T*) is the rate constant of the reduction reaction at temperature *T*; *S*(*τ*), *s*(*τ*) are absolute and specific “active” surface areas of the metal oxide at time *τ*; *n*(*τ*) is the molar amount of the substance at time *τ*; *P*_g_ is the hydrogen partial pressure in the system. Conversion degree *α* is connected with molar content *via* the expression:

5 *n* = *n*_0_(1 − *α*)

Substituting (5) into (4) and taking into account that *α* is a single-valued function of *τ* we get the following equation:6
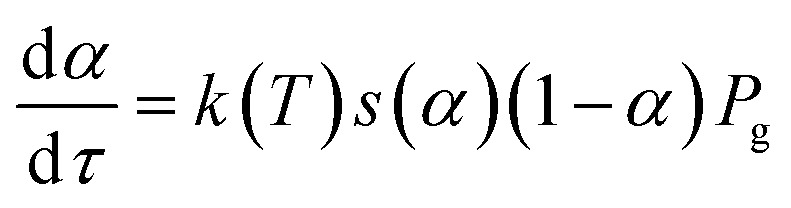


Comparing eqn (6) and (1), and assuming *k*(*T*) = A* exp(−*E*_a_/*RT*), we find that:

7 *f*(*α*) = *s*(*α*)(1 − *α*)*P*_g_

Thus, the conversion function in that case is connected with the function of specific surface area change; *i.e.* if *f*(*α*) is known, then the *s*(*α*) can be evaluated and *vice versa*. At the present time, there are plenty of mathematical forms to express *f*(*α*).^24^ The choice of a certain type of conversion function is based either on *a priori* knowledge about the geometry of the interfacial reacting surface or just on the lowest residual dispersion between the theoretical and experimental curves. However, in both cases the chosen conversion function far from always reflects the real surface geometry of the investigated system: *a priori* considerations can be wrong; function choice based on minimal residual dispersion also can be wrong if none of the test functions describe the surface geometry. In connection with this, we suggest another approach to the choice of optimal conversion function. The idea is simple: instead of determination of what known function exactly corresponds to the investigated system, we evaluate the optimal function of the surface area change, corresponding to the investigated TPR curves. Further, the realization of the suggested approach will be given taking into account experimental parameters (gas flow rate, hydrogen concentration in the gaseous mixture, heating rate *etc.*).

### 3.2 Calculations

Consider the kinetics of an *n*-staged reduction of some oxide (Ox_0_) by hydrogen to metal state (Me) in the process of TPR:8
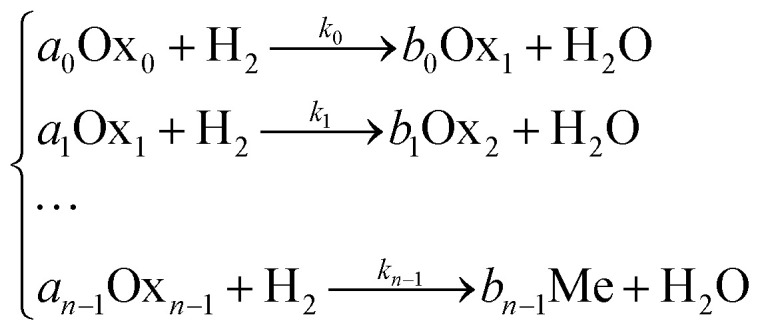
9
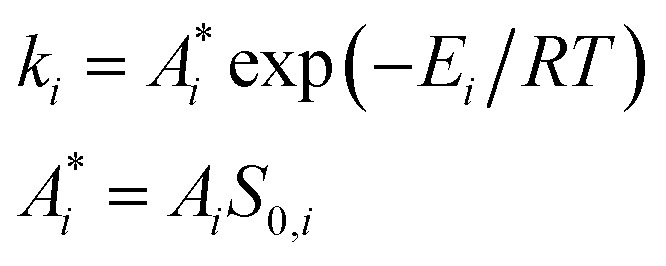
where *k*_*i*_ is the rate constant of the *i*^th^ stage of reduction; is the apparent preexponential factor (preexponential factor multiplied by the specific surface area (*S*_0,*i*_) of the oxide at zero conversion); *E*_*i*_ is the activation energy of the *i*^th^ stage.

Within the small time interval that corresponds to the time needed to fill the tube with the gaseous mixture, when the temperature change and convective transfer can be neglected, the process is described by the following system of differential equations:10
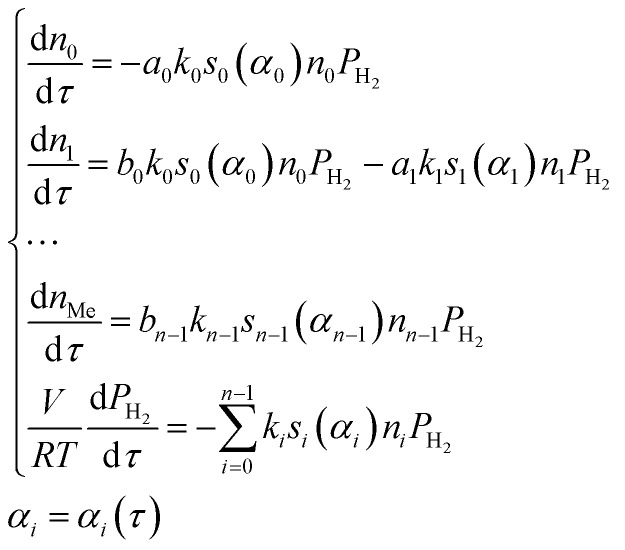
where *α*_*i*_ is the degree of conversion of *i*^th^ oxide; *s*_*i*_(*α*_*i*_) is the function representing the dependence of the specific relative surface area of the *i*^th^ oxide from its degree of conversion; *V* is the reactor volume, containing reduced oxide. In order to solve this system of equations within the certain time interval, one needs to know *n* pairs of values of 
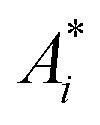
, *E*_*i*_ and *n* functions of *s*_*i*_(*α*_*i*_). Let all the *s*_*i*_(*α*_*i*_) functions belong to one class and differ by only some vector of values *λ*_*i*_(*y*_*i*,0_, …, *y*_*i*,*m*−1_) of length *m*. Thus, if we know *n* pairs of values 
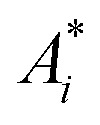
, *E*_*i*_ and n*m values of *y*_*ij*_, modeling of the TPR curve in the process of Ox_0_ reduction can be carried out in the following way.

Let's introduce the modeling step, which corresponds to the time interval needed to fill the reactor's volume with the gaseous mixture. For a tubular reactor we have:11
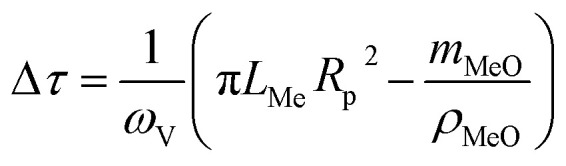
where *ω*_V_ is the gas flow rate; *L*_Me_ is the length of the tube segment with sample; *R*_p_ is the tube radii; *m*_MeO_ and *ρ*_MeO_ are the sample mass and density, respectively.

We propose that within the time interval Δ*τ*, the temperature does not change and equals:12
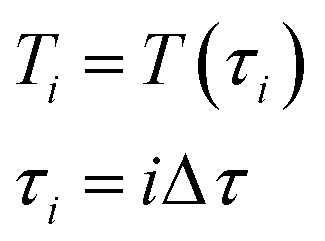


After Δ*τ* has passed, the temperature changes abruptly up to the value *T*_*i*+1_ = *T*(*τ*_*i*+1_) = *T*(*τ*_*i*_ + Δ*τ*). On every time interval, the system of differential equations is solved numerically, and the change in hydrogen partial pressure and the composition of the reactive mixture are estimated. During the next-time step, the reaction mixture is filled with a new portion of gaseous mixture and the process of modeling is repeated. Because Δ*τ* is small, such a scheme allows the avoidance of a numerical solution of a differential equation of convective transfer and, at the same time, provides sufficient accuracy of the modeling (taken from preliminary numerical experiments). As a result, the model TPR is built:13
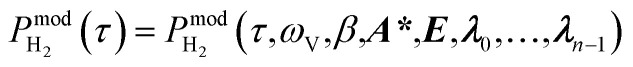


Because the time step is small, the obtained model curve can be considered as continuous. To solve the inverse problem; *i.e.* to identify the set of parameters A*,**E**,**λ**_0_,…,**λ**_*n*−1_ that allow such model curves to fit the experimental curves (with various heating rates) as best as possible, one has to minimize the following function:14
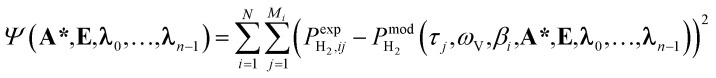
where 
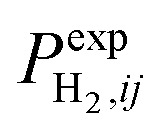
 is the hydrogen partial pressure at the *j*^th^ point of the experimental curve with *i*^th^ heating rate. In order to solve this problem, one needs to know the certain type of function *s*(*α*, *λ*). All that is known about it is that *s*(*α* = 0, *λ*) ≡ 1.0. Because any other information about this function is absent (other than it should be continuous, single valued and limited in its domain (0.0; 1.0)), in present work we suggest it is sought in the class of cubic splines.^25^ At the present time, cubic splines are successfully used for the interpolation and approximation of various functions and experimental data. Cubic spline with *m* knots is determined by a set of *m* + 2 parameters; *i.e. m* values in spline knots and two parameters, defining the type of the cubic spline. Usually, these two additional parameters are values of the second derivative of the spline in the first and in the last knot (if one takes this value to equal 0 it will result in a natural cubic spline). However, in the present work, these two parameters were used to build the cubic spline with the minimum norm of the first derivative, as in ref. 26. Spline with a minimum norm of the first derivative differs from the ordinary one by the absence of oscillations of values in between the knots, that is in agreement with the most general assumptions about the character of dependence of specific surface area from conversion. Finally, in our work to describe *s*(*α*, *λ*), the cubic splines with minimum norm of the first derivative have been used. Also, *λ* = (*y*_1_, …, *y*_*m*−1_) for the spline with *m* knots; *i.e. m*−1 value in spline knots, because the value in the first knot (*y*_0_) is always 1. To minimize (14) it is better to use methods of nonlinear minimization of zero order that do not require derivative evaluation, because analytical expression for the object function is unknown (the system of differential eqn (10) is solved numerically) and using numerical approximation of derivatives drastically reduces the efficiency of methods similar to Newton's. After a series of numerical experiments in the present work to minimize (14), we used the Covariance Matrix Adaptation Evolution Strategy (CMA-ES) method.^27^ This method is of zero order, possesses good convergence and is undemanding in terms of the choice of initial approximation. Preliminary calculations showed that using the object function mentioned above for the minimization allowed no stable solutions. Such behavior is typical for ill-posed problems. In order to solve such problems, regularization methods are usually used.^28^ It is known that the formation of the new interface surface requires some energy; namely Δ*E*_S_ = *σ*Δ*S*, where *σ* is surface tension and Δ*S* is surface change. Because spontaneous processes progress toward an energy minimum, we presume it is logical to use the value of a general increase of the interfacial surface in the process of oxide reduction as a stabilizer. Therefore, for some oxides:15
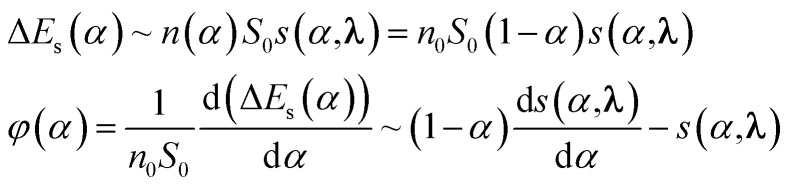


Let's introduce new function *φ*^*+*^(*α*) as the following:16
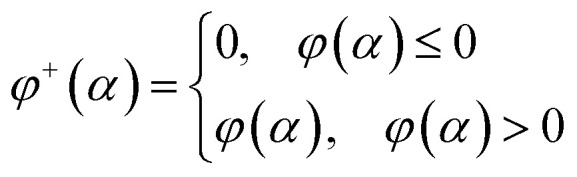


The following function is used as a stabilizer in the present work:17
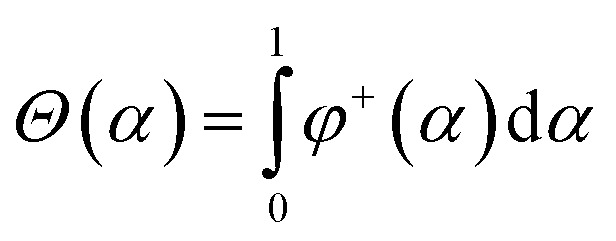


Finally, the object function (14) is transformed into:18
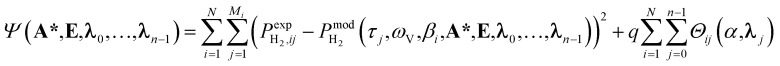
where *q* is a regularization parameter.

After we obtained an optimal set of kinetic parameters and splines of relative specific surface area we tried to evaluate the initial particle size distribution, *ω*, based on the assumption that initial particle size distribution defines explicitly how relative specific surface area changes throughout the process of TPR. We take into account only the initial iron oxide, Fe_2_O_3_, because its reduction is characterized by the process of contraction of the particles during TPR, while the other oxides proceed *via* the growth and contraction stages that cannot be evaluated and separated precisely. In order to obtain an initial particle size distribution using the optimal *s*(*α*) spline and *n*(*α*) dependence, we conducted an iterative procedure until the particle size distribution on the *n*th step reproduces the optimal *s*(*α*) spline. The following iterative formula was used:19
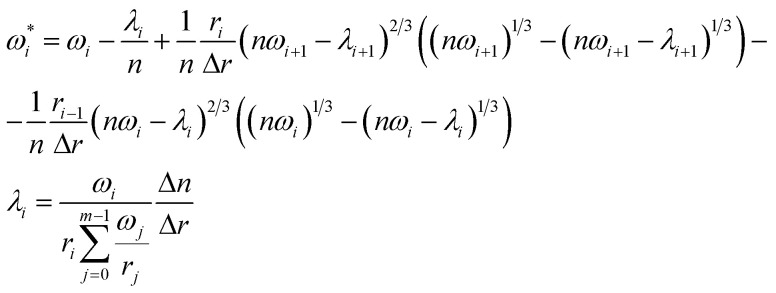
where 
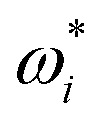
 and *ω*_*i*_ are the particle size distribution values of the *i*^*t*h^ size interval on the next and current step, respectively; *n* and Δ*n* are the total mole amount and its change; 
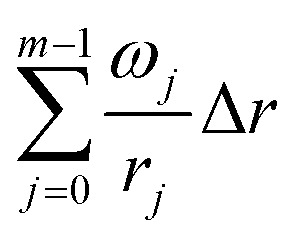
 is the normalization factor; *r* and Δ*r* are the size and width of the size interval. The full derivation can be found in the ESI.†

In present work, we conducted research on the applicability of the suggested method to describe the kinetics of a heterogeneous chemical reaction on the example of TPR of iron(iii) oxide. We provide the results of calculations and compare them with the corresponding experimental data.

## 4 Results and discussion

The reduction process of iron(iii) oxide has been studied in depth^29–34^ due to the wide scope of applications of iron and steel in modern technologies. Thus, iron-based catalysts are prospective because of their low price and low methane selectivity in the Fischer–Tropsch process.^12,35,36^

**Fig. 1:**
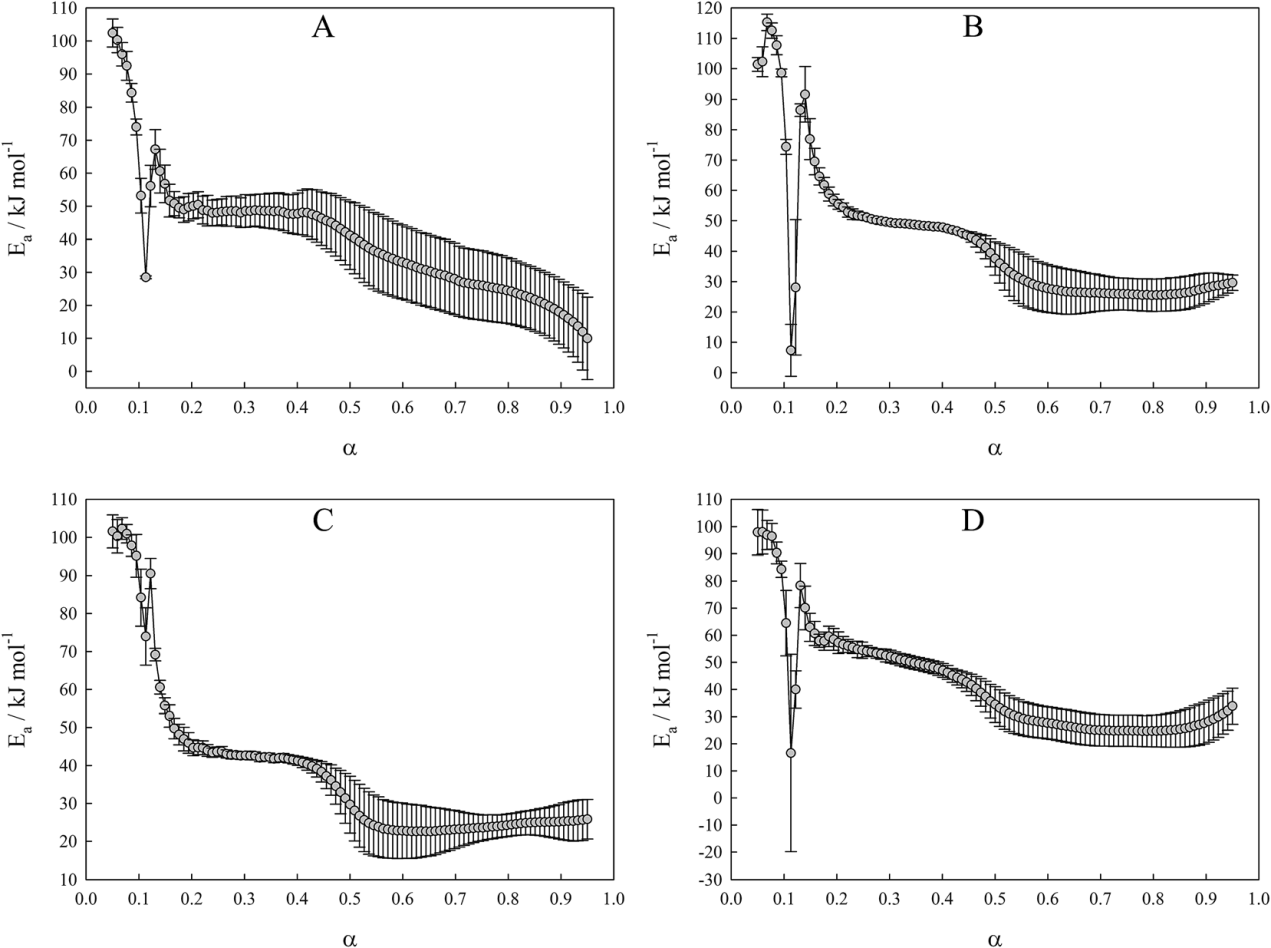
Dependence of activation energy of iron(iii) oxide reduction from conversion degree, calculated *via* Friedman method. (A) Original Fe_2_O_3_ powder; (B) Fe_2_O_3_ annealed at 600 °C; (C) Fe_2_O_3_ annealed at 700 °C; (D) Fe_2_O_3_ annealed at 800 °C.

TPR of iron(iii) oxide is very complicated in terms of kinetic analysis because of the multistage character of the reduction (Fe_2_O_3_ → Fe_3_O_4_ → FeO → Fe).^37^ Every stage of this reduction process is characterized by a separate set of kinetic parameters that makes model-free methods, based on eqn (1), inapplicable to the kinetic analysis of such a process. This is also proved by the wide range of activation energies (18–246 kJ mol^−1^)^32^ caused not only by the various compositions and morphologies of the investigated sample, but also by inaccurate kinetic analysis. In our work we used the Friedman method to obtain dependence *E*_a_ from *α* in order to compare it with the corresponding values evaluated *via* the suggested method. The obtained curves (Fig. 1) clearly demonstrate that the reduction passes through three consecutive steps as there are three regions on the Friedman plots, where *E*_a_ values remain more or less constant. The disadvantage of the Friedman method in this case is that *E*_a_ cannot be evaluated precisely for each stage due its variation with respect to the studied material or the degree of conversion. In particular, the activation energy of the first reduction step Fe_2_O_3_–Fe_3_O_4_, proceeding while the conversion degree has not reached 0.11, lies in the range from 7 to 115 kJ mol^−1^, therefore making evaluation of this parameter inaccurate. Such a wide confidence interval occurs because the reduction of each oxide contributes to the d*α/*d*t* term. Because reduction reactions proceed with different rates, the contribution of each reduction reaction to the d*α/*d*t* term varies when the heating rate changes, thus widening the interval of the activation energy values. This complication arises from eqn (1), which can be utilized properly only in the case of a single-stage processes. Also, it can be seen that the activation energies of the other reduction stages oscillate from one curve to another, thus suggesting that the single-rate eqn (1) cannot be applied to a multistage process.

In our study we used samples of different morphologies to assess the variation in the kinetic analysis results with respect to the studied material. To ensure that all samples have identical chemical composition, we annealed iron(iii) oxide at different temperatures to obtain series of samples with different morphologies.

**Fig. 2:**
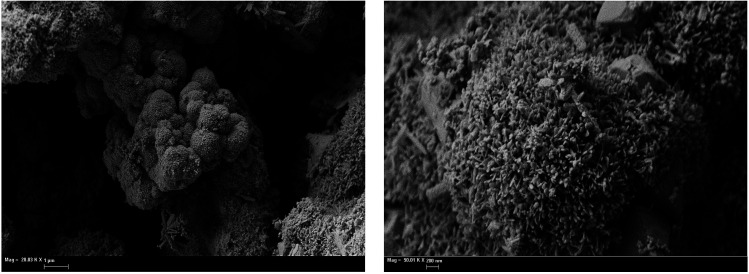
SEM images of the original Fe_2_O_3_ powder.

SEM images of the initial iron oxide powder are shown in Fig. 2. The original powder consists of spherical particles aggregated from flakes of Fe_2_O_3_. This sample possesses a highly-developed surface structure, proved by the highest BET surface area among all studied materials (25 m^2^ g^−1^; Table 1).

**Table 1:** Optimized values of activation energy and preexponential factor for the reduction of iron oxide(iii) samples and BET surface area values

Sample	*E*_Fe_2_O_3__/kJ mol^−1^	*E*_Fe_3_O_4__/kJ mol^−1^	*E*_FeO_/kJ mol^−1^	*A*_Fe_2_O_3__/s^−1^	*S*_spec._/m^2^ g^−1^
Fe_2_O_3_ original powder	114.1	98.8	72.9	304.9	25.0
Fe_2_O_3_ annealed at 600 °C	120.0	111.7	72.2	264.7	4.9
Fe_2_O_3_ annealed at 700 °C	111.8	115.9	73.6	44.8	4.4
Fe_2_O_3_ annealed at 800 °C	104.3	113.9	83.9	7.9	3.0

**Fig. 3:**
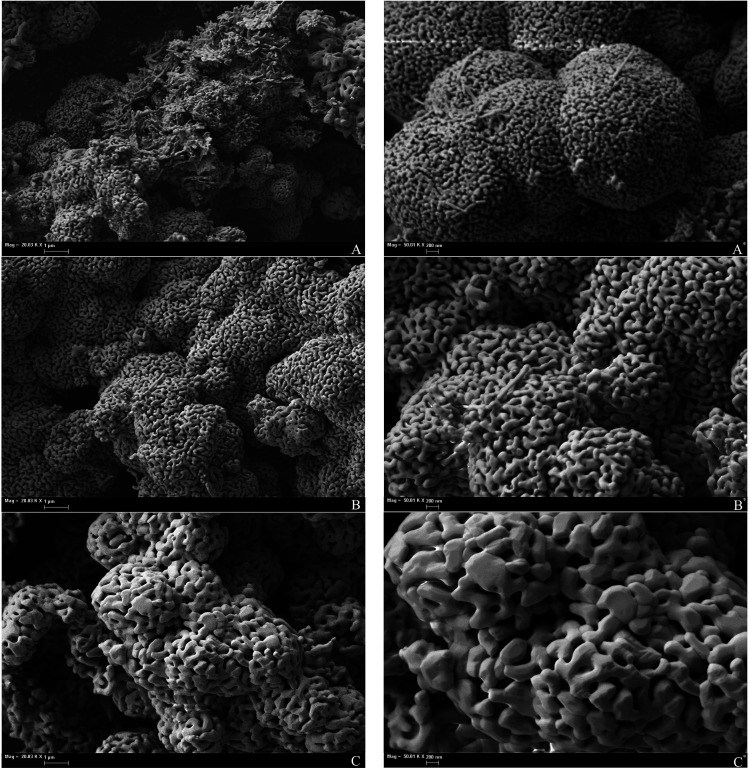
SEM images of the Fe_2_O_3_ samples, annealed at various temperature. (A) 600 °C; (B) 700 °C; (C) 800 °C.

Annealing of the iron oxide powder drastically changes the surface morphology of the material. SEM images of annealed samples are given in Fig. 3. Annealed oxides have a labyrinth structure and become more amorphous from the surface with growth of annealing temperature. Increasing annealing temperature leads to particle growth and to a decrease of the inner particle pores that were in original powder (Fig. 2 and 3). At 800 °C, particles tend to sinter into larger grains and the number of surface pores became minimal (Fig. 3C). Specific surface area of the annealed samples decreases with increasing annealing temperature (4.9, 4.4 and 3.0 m^2^ g^−1^; Table 1). Although values of the specific surface area of annealed samples are close to each other, such small differences in morphology affects the TPR spectrum and can be revealed by the analysis of optimized kinetic parameters, as discussed below.

**Fig. 4:**
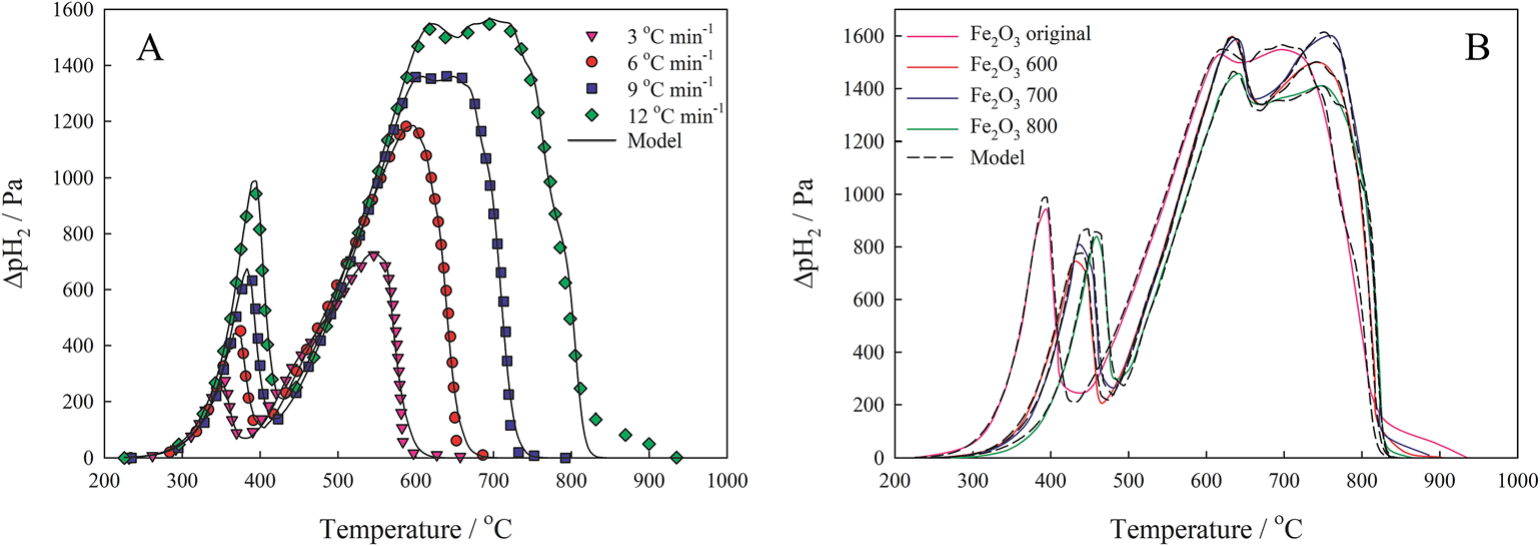
TPR curves of the original Fe_2_O_3_ powder (A) recorded under various heating rates, and TPR curves of iron(iii) oxide samples (B) recorded under 12 °C min^−1^ heating rate.

TPR curves of iron(iii) oxide samples, recorded at several heating rates (3, 6, 9 and 12 °C min^−1^), are presented in Fig. 4A. Using data obtained at several temperature regimes improves the reliability of the results of the kinetic analysis according to the International Congress on Thermal Analysis and Calorimetry (ICTAC) project recommendations.^38^ Curve fitting was carried out using a three-stage scheme of reduction, with the following kinetic equations:20
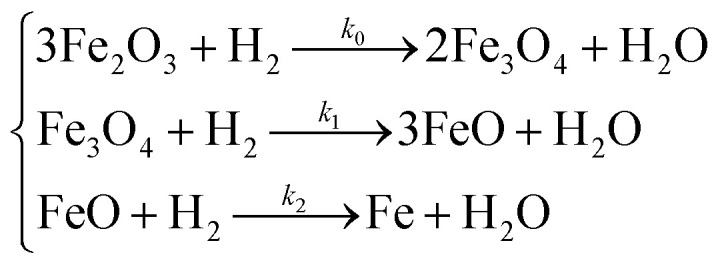
21
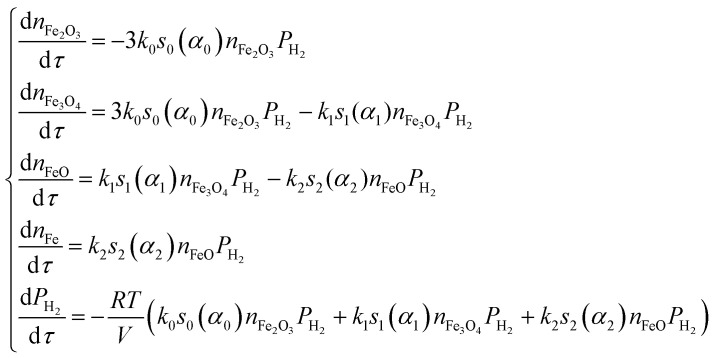


The shape of the reduction curve changes with heating rate, with process shifts to higher temperatures (Fig. 4A). The first reduction peak shifts by 50 °C from 3 °C min^−1^ curve to 12 °C min^−1^. There are also variations in TPR curves if we consider them in the row of studied materials (Fig. 4B). The first maximum in the TPR of iron(iii) oxides also shifts to higher temperatures in the progression from original powder to the most annealed. This clearly demonstrates the influence of the surface morphology of the investigated samples on the TPR spectra and, as a result, on the kinetic analysis. However, all TPR curves are approximated well, and the optimization procedure resulted in close values of kinetic parameters demonstrating high reliability of the suggested method (Table 1). It is noteworthy that the preexponential factors found for the first step of reduction correlate well with the specific surface areas of the materials. If we recall that the preexponential factor is a multiplication of the specific surface area at zero conversion and true preexponential factor (9) at a given temperature, then it should correlate to some extent with the specific surface area. Although there is a loose correlation between them, because we deal with the “active” specific surface area that is not the one measured by nitrogen, preexponential factors form the same dependence as the specific surface areas (*A*_1_ > *A*_2_ > *A*_3_ > *A*_4_ as well as *S*_1_ > *S*_2_ > *S*_3_ > *S*_4_). Thus, we can conclude that the suggested method takes into account the difference in material morphology and is capable of providing reliable kinetic results.

**Fig. 5:**
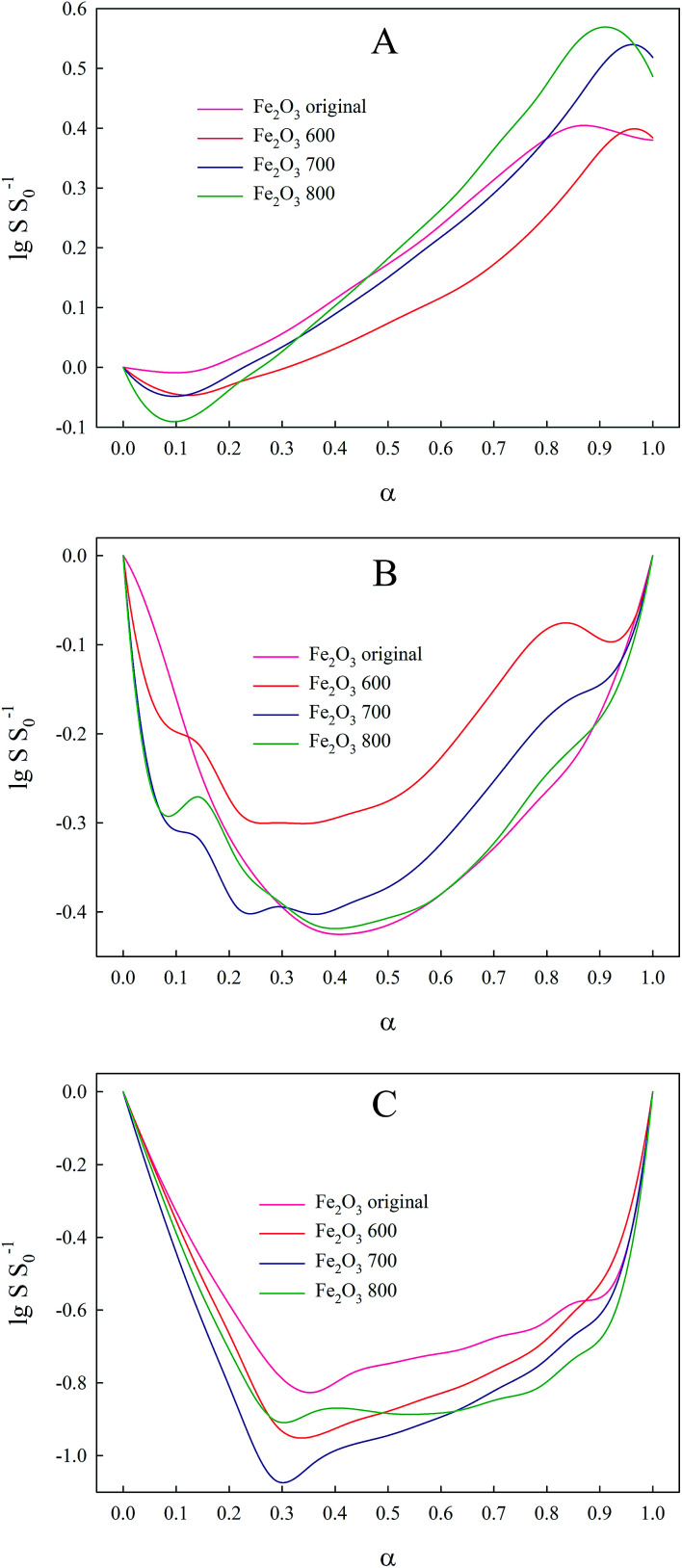
Optimized relative specific surface area *vs.* conversion degree curves of oxide forms obtained for 12 °C min^−1^ heating rate. (A) Fe_2_O_3_; (B) Fe_3_O_4_; (C) FeO.

Besides kinetic parameters, we also obtained *s*(*α*) curves as presented in Fig. 5. Change in relative specific surface area with degree of conversion for Fe_2_O_3_ oxide is a monotone increasing function (Fig. 5A) that can be explained by the contraction particle model. When particles of hematite react with hydrogen, their size decreases and at the same time the specific surface area of the particles increases due to the fact that the ratio between the surface atoms and the bulk atoms grows. The shape of the *s*(*α*) curves of the intermediate oxides Fe_3_O_4_ and FeO (Fig. 5B and C) is caused by the same factors. The first particles of Fe_3_O_4_ formed from the reduced hematite began to grow; thus, their specific surface area decreases. Then, when the reduction rate of Fe_3_O_4_ exceeds the growth of Fe_3_O_4_, the relative specific surface area begins to grow. It is for this reason that we observe a minimum on the *s*(*α*) curve. The same hypothesis we be applied for *s*(*α*) dependence of FeO.

**Fig. 6:**
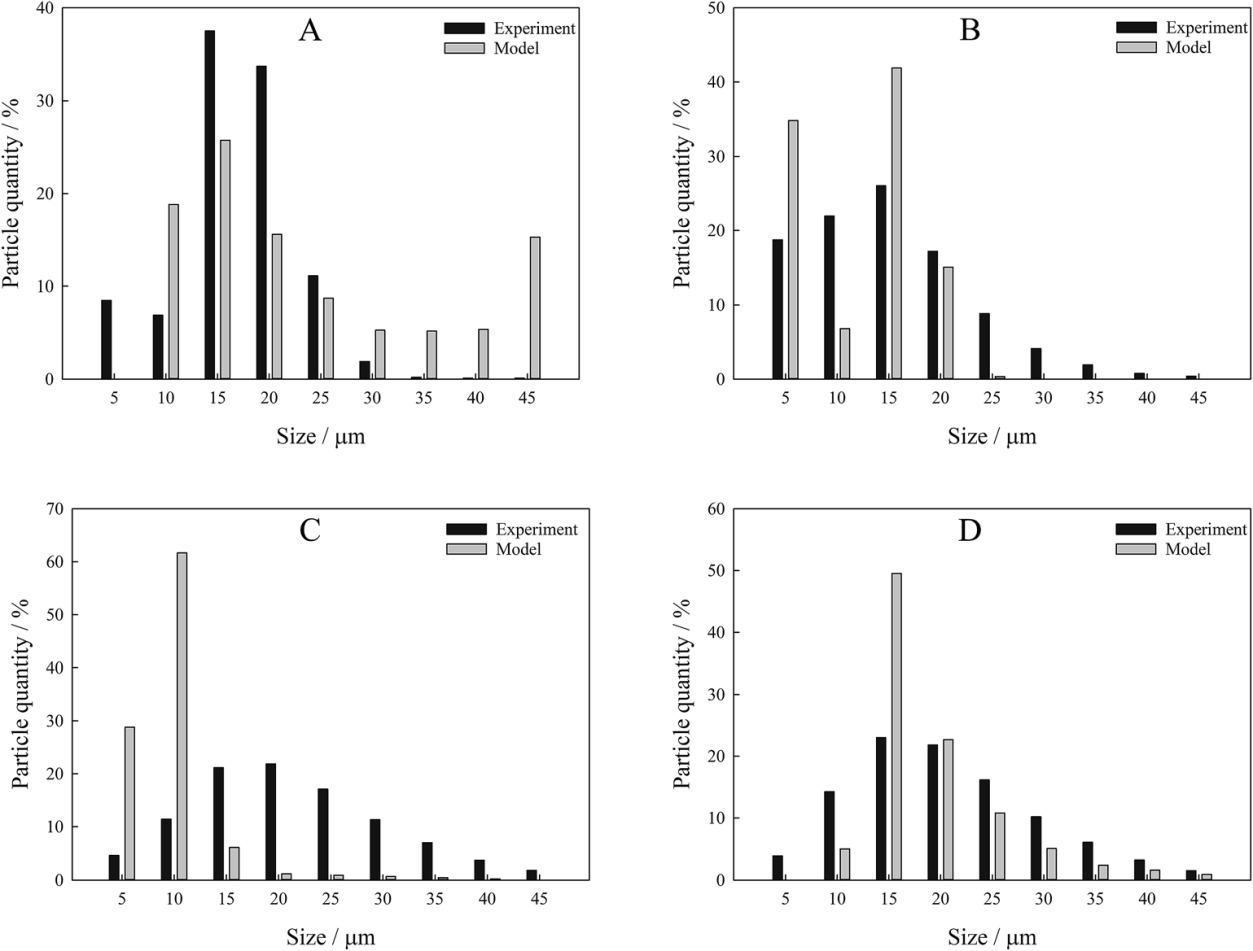
Particle size distribution bar charts of the studied samples obtained *via* iterative procedure and experimentally measured. (A) Original Fe_2_O_3_ powder; (B) Fe_2_O_3_ annealed at 600 °C; (C) Fe_2_O_3_ annealed at 700 °C; (D) Fe_2_O_3_ annealed at 800 °C.

From *s*(*α*) of Fe_2_O_3_, we reproduce the initial particle size distribution using the iterative procedure described in the Calculations section. We evaluated particle size distribution and compared it with the one measured experimentally using a laser diffraction method (Fig. 6). The results have shown that the proposed method allows accurate reproduction of the particle sizes, in particular, the mode in particle size distribution based on very simple theoretical assumptions. Deviations of the model from the experimental values come from the fact that the procedure of nonlinear minimization used in the work is very sensitive to the initial values of the optimized parameters. Good consistency between the model and the theory proves that the suggested method for kinetic analysis of TPR satisfies all the requirements and can be used in wider research practice.

## 5 Conclusions

A new approach to the kinetic analysis of the kinetics of heterogeneous reactions in terms of a TPR has been suggested. The method is based on the optimization of activation energy and the preexponential factor separately from the relative surface area function and evaluating this function as cubic spline, which allows non-isothermal data to be described with controlled precision depending on the number of spline knots. Analysis of the optimized relative surface area function enables one to qualitatively assess the influence of temperature and heating rate on the kinetics of the heterogeneous chemical processes.

The presented approach was implemented in the kinetic analysis of the TPR of iron(iii) oxide samples that possess different surface morphology under different heating rates (3–12 °C min^−1^). Close values of kinetic parameters were obtained for the studied materials by the presented method, proving its reliability. Correlation between the found preexponential factors and the specific surface areas of the iron(iii) oxide samples demonstrates the capability of the method to probe morphology variation among the series of studied materials (from the original Fe_2_O_3_ sample to the sample annealed at 800 °C: A* – 304.9 > 264.7 > 44.8 > 7.9; *S*_spec._ – 25.0 > 4.9 > 4.4 > 3.0). Using optimized *s*(*α*) dependence, the initial particle size distribution of the sample was obtained and compared with the experimental distribution obtained *via* laser diffraction. Deviations between model and experimental values can be attributed to the strong dependence of the results from the initial values of the optimized parameters.

## Conflicts of interest

There are no conflicts to declare.

## Supplementary Material

RA-008-C7RA09848K-s001

## References

[cit1] Budrugeac P., Segal E. (2000). Polym. Degrad. Stab..

[cit2] Hatakeyama T. and Quinn F. X., Thermal Analysis: Fundamentals and Applications to Polymer Science, John Wiley and Sons, Chichester, 1999, 2nd edn

[cit3] Wunderlich B. , Thermal Analysis of Polymeric Materials, Springer, Berlin, 2005

[cit4] Jones A. and McNicol B. D., Temperature-programmed reduction for solid materials characterization, Marcel Dekker, New York, 1986

[cit5] Kanervo J. M., Krause A. O. I. (2002). J. Catal..

[cit6] Gervasini A. , Temperature Programmed Reduction/Oxidation (TPR/TPO) Methods, in Calorimetry and Thermal Methods in Catalysis, ed. A. Auroux, Springer, 2013, pp. 175–197

[cit7] Huang Q., Yan X., Li B., Xu X., Chen Y., Zhu S., Shen S. (2013). J. Ind. Eng. Chem..

[cit8] Zhang Y., Qin Z., Wang G., Zhu H., Dong M., Li S., Wu Z., Li Z., Wu Z., Zhang J., Hu T., Fan W., Wang J. (2013). Appl. Catal., B.

[cit9] Zhang C.-H., Wan H.-J., Yang Y., Xiang H.-W., Li Y.-W. (2006). Catal. Commun..

[cit10] Yiu H. H. P., Keane M. A., Lethbridge Z. A., Lees M. R., El Haj A. J., Dobson J. (2008). Nanotechnology.

[cit11] Mogorosi R. P., Fischer N., Claeys M., van Steen E. (2012). J. Catal..

[cit12] Picassoa G., Cruza R., Sun Kou M. D. R. (2015). Mater. Res. Bull..

[cit13] Jothimurugesan K., Goodwin J. G., Gangwal S. K., Spivey J. J. (2000). Catal. Today.

[cit14] Khzouz M., Wood J., Pollet B., Bujalski W. (2013). Int. J. Hydrog. Energy.

[cit15] Sokolov S., Kondratenko E. V., Pohl M.-M., Barkschat A., Rodemerck U. (2012). Appl. Catal., B.

[cit16] Vyazovkin S., Wight C. A. (1997). Annu. Rev. Phys. Chem..

[cit17] Budrugeac P. (2013). Thermochim. Acta.

[cit18] Friedman H. L. J. (1972). J. Polym. Sci. B.

[cit19] Flynn J. H., Wall L. A. (1996). J. Res. Natl. Bur. Stand., Sect. A.

[cit20] Doyle C. D. (1962). J. Appl. Polym. Sci..

[cit21] Vyazovkin S., Burnham A. K., Criado J. M., Pérez-Maqueda L. A., Popescu C., Sbirrazzuoli N. (2011). Thermochim. Acta.

[cit22] Koga N. (1994). Thermochim. Acta.

[cit23] Brown M. E., Galwey A. K. (2002). Thermochim. Acta.

[cit24] Vyazovkin S. (2016). Phys. Chem. Chem. Phys..

[cit25] Fan J. and Yao Q., Nonlinear Time Series: Nonparametric and Parametric Methods, Springer-Verlag, New York, 2003

[cit26] Dmitriev V. I., Ingtem Z. G. (2010). Computational Mathematics and Modeling.

[cit27] Hansen N., Ostermeier A. (2001). Evol. Comput..

[cit28] Tikhonov A. N. , Goncharsky A. V., Stepanov V. V. and Yagola A. G., Numerical Methods for the Solution of Ill-Posed Problems, Kluwer Academic Publishers, 1995

[cit29] Maiti G. C., Lochner U., Baerns M. (1987). Thermochim. Acta.

[cit30] Lin H.-Y., Chen Y.-W., Li C. (2003). Thermochim. Acta.

[cit31] Pourghahramani P., Forssberg E. (2007). Thermochim. Acta.

[cit32] Pineau A., Kanari N., Gaballah I. (2006). Thermochim. Acta.

[cit33] Pineau A., Kanari N., Gaballah I. (2007). Thermochim. Acta.

[cit34] Shanker ray H., Kundu N. (1986). Thermochim. Acta.

[cit35] Pham H. N., Datye A. K. (2000). Catal. Today.

[cit36] Bukur D. B., Nowicki L., Manne R. K., Lang X. (1995). J. Catal..

[cit37] Jozwiak W. K., Kaczmarek E., Maniecki T. P., Ignaczak W., Maniukiewics W. (2007). Appl. Catal., A.

[cit38] Vyazovkin S., Chrissafis K., Di Lorenzo M. L., Koga N., Pijolat M., Roduit B., Sbirrazzuoli N., Suñol J. J. (2014). Thermochim. Acta.

