# Supplementary material for: An alternative approach to kinetic analysis of temperature-programmed reaction data
Source: RSC Adv. 2018 Jan 16;8(6):3286–95. doi: 10.1039/c7ra09848k (PMC9077687; doi:10.1039/c7ra09848k)
Supplement: RA-008-C7RA09848K-s001 [file RA-008-C7RA09848K-s001.pdf]

Electronic Supplementary Material (ESI) for RSC Advances.
This journal is © The Royal Society of Chemistry 2018

Particle size distribution estimation. Absolute size scale

Investigated sample in temperature-programmed reduction (TPR) method is a powder. In our case it is a powder of chemically pure substance consisting of separate particles of undefined shape are distributed with respect to characteristic sizes (further – sizes). Characteristic size is a value, which square is proportional to the surface area and its cube is proportional to the particle's volume (for spherical particles characteristic size is radius, for cubic – cube edge and etc.). Each particle is a solid phase of a certain size, while interaction with gaseous agent can occur only on the surface of this particle (i.e. diffusion into the particle is either impossible or negligible compared to the rate of heterogeneous reaction). Let's introduce discrete size distribution (from r_{min} to r_{max}) denoted as ω. For n moles of substance the number of particles with size r_i will be:

$$n_i = n\omega_i \Delta r \qquad \text{* MERGEFORMAT (1)}$$

Their volume can be written as:

$$V_i = \frac{n_i}{\rho} = \frac{n}{\rho}\omega_i \Delta r \qquad \text{* MERGEFORMAT (2)}$$

$\rho -$ substance's molar density. Volume and surface area of one particle are:

$$v_i = f_v r_i^3, \quad s_i = f_s r_i^2 \qquad \text{* MERGEFORMAT (3)}$$

$f_v, f_s -$ coefficients depending on particle's shape (for spheres: $f_s = 4\pi$, $f_v = 4/3\pi$). Then the number of particles with size r_i:

$$N_i = \frac{V_i}{v_i} = \frac{n}{\rho}\frac{\omega_i \Delta r}{f_v r_i^3} \qquad \text{* MERGEFORMAT (4)}$$

Their total surface area is:

$$S_i = N_i s_i = \frac{f_s}{f_v}\frac{n}{\rho}\frac{\omega_i}{r_i}\Delta r \qquad \text{* MERGEFORMAT (5)}$$

Total surface area of n moles of substance:

$$S_\Sigma = \sum_{i=0}^{m-1} S_i = \frac{f_s}{f_v}\frac{n}{\rho}\Delta r \sum_{i=0}^{m-1}\frac{\omega_i}{r_i} \qquad \text{* MERGEFORMAT (6)}$$

Specific molar surface area:

$$\overline{S} = \frac{S_\Sigma}{n} = \frac{f_s}{f_v}\frac{1}{\rho}\Delta r \sum_{i=0}^{m-1}\frac{\omega_i}{r_i} \qquad \text{* MERGEFORMAT (7)}$$

Relative specific surface area:

$$\overline{S}_r = \frac{\overline{S}}{\overline{S}_0} = \frac{\sum_{i=0}^{m-1}\frac{\omega_i}{r_i}\Delta r}{\sum_{i=0}^{m-1}\frac{\omega_i^0}{r_i}\Delta r} \qquad \text{* MERGEFORMAT (8)}$$

For TPR of oxides if only one oxide presented in the system at the process beginning and other are generated in the process of reduction, then in the moment of their formation:

$$\omega_0^0 = 1/\Delta r,\ \omega_i^0\Big|_{i>0} = 0 \qquad \text{* MERGEFORMAT (9)}$$

Because in the beginning of the process the oxide's nuclei are formed with minimal possible particle size. Then we obtain:

$$\overline{S}_0 = \frac{f_s}{f_v}\frac{1}{\rho}\sum_{i=0}^{m-1}\frac{\omega_i^0}{r_i}\Delta r = \frac{f_s}{f_v}\frac{1}{\rho r_{\min}} \qquad \text{* MERGEFORMAT (10)}$$

That's why for relative specific surface area of all intermediate oxide could be written as:

$$\overline{S}_r = r_{\min}\sum_{i=0}^{m-1}\frac{\omega_i}{r_i}\Delta r \qquad \text{* MERGEFORMAT (11)}$$

Let's postulate that any powder sample has only one particle of the maximal size. It is obvious that this is the only particle in the last size interval. Then:

$$n_{\max} = An\omega_{m-1}\Delta r \qquad \text{* MERGEFORMAT (12)}$$

$n_{\max}$ – molar quantity of the substance in the particle of the largest size; ω – unnormalized density function; A – normalizing factor ($A\Delta r\sum_{i=0}^{m-1}\omega_i = 1$). If we consider particles to have spherical shape, then:

$$r_{\max} = \left(n_{\max}\frac{3}{4\pi}\frac{1}{\rho}\right)^{\frac{1}{3}} = \left(An\omega_{m-1}\Delta r\frac{3}{4\pi}\frac{1}{\rho}\right)^{\frac{1}{3}} \qquad \text{* MERGEFORMAT (13)}$$

$r_{\max}$ – particle's radius. Minmal radius will be:

$$r_{\min} = r_{\max}/m = \frac{1}{m}\left(An\omega_{m-1}\Delta r\frac{3}{4\pi}\frac{1}{\rho}\right)^{\frac{1}{3}} \qquad \text{* MERGEFORMAT (14)}$$

Step size Δr can be found from the ratio:

$$r_{\min} + (m-1)\Delta r = r_{\max}$$

$$\Delta r = \frac{r_{\max} - r_{\min}}{m-1}$$

$$r_{\max} = \left(n_{\max}\frac{3}{4\pi}\frac{1}{\rho}\right)^{\frac{1}{3}} = \left(An\omega_{m-1}\Delta r\frac{3}{4\pi}\frac{1}{\rho}\right)^{\frac{1}{3}}$$

$$r_{\min} = r_{\max}/m = \frac{1}{m}\left(An\omega_{m-1}\Delta r\frac{3}{4\pi}\frac{1}{\rho}\right)^{\frac{1}{3}}$$

$$\Delta r = \frac{r_{\max} - r_{\min}}{m-1} = \frac{1}{m-1}\left(\left(An\omega_{m-1}\Delta r\frac{3}{4\pi}\frac{1}{\rho}\right)^{\frac{1}{3}} - \frac{1}{m}\left(An\omega_{m-1}\Delta r\frac{3}{4\pi}\frac{1}{\rho}\right)^{\frac{1}{3}}\right)$$

$$\Delta r^{2/3} = \frac{1}{m}\left(An\omega_{m-1}\frac{3}{4\pi}\frac{1}{\rho}\right)^{\frac{1}{3}} \qquad \text{* MERGEFO}$$

$$\Delta r = \frac{1}{m^{3/2}}\left(An\omega_{m-1}\frac{3}{4\pi}\frac{1}{\rho}\right)^{\frac{1}{2}}$$

Normalizing condition:

$$A\Delta r\sum_{i=0}^{m-1}\omega_i = 1 \quad \text{* MERGEFORMAT (16)}$$

Substituting * MERGEFORMAT (15) into * MERGEFORMAT (16), leads to:

$$A\frac{1}{m^{3/2}}\left(An\omega_{m-1}\frac{3}{4\pi}\frac{1}{\rho}\right)^{\frac{1}{2}}\sum_{i=0}^{m-1}\omega_i = 1$$

$$A^{3/2}\frac{1}{m^{3/2}}\left(n\omega_{m-1}\frac{3}{4\pi}\frac{1}{\rho}\right)^{\frac{1}{2}}\sum_{i=0}^{m-1}\omega_i = 1$$

$$A^{3/2} = \frac{m^{3/2}}{\left(n\omega_{m-1}\frac{3}{4\pi}\frac{1}{\rho}\right)^{\frac{1}{2}}\sum_{i=0}^{m-1}\omega_i} \quad \text{* MERGEFORMAT (17)}$$

$$A = \frac{m}{\left(n\omega_{m-1}\frac{3}{4\pi}\frac{1}{\rho}\right)^{1/3}\left(\sum_{i=0}^{m-1}\omega_i\right)^{2/3}}$$

Thus:

$$r_i = r_{\min} + i\Delta r, 0 \le i \le m-1 \quad \text{* MERGEFORMAT (18)}$$

Let’s consider that as a result of some heterogeneous reaction between powder and gaseous agent the molar quantity of solid phase reduced by Δn moles. Because rate of heterogeneous reaction is proportional to area of the interface, the quantity of substance removed from the particle will proportional to surface of this particle. While the contraction of the particle size occurs and, therefore, the transformation of size density function. After substance removal size of all particles in every size interval will be reduced by the same value, while their quantity remains constant. Thus, new particle size can be evaluated and, therefore, new interval (in particular, two neighboring intervals) that correspond to the reduced particles. Initial particles quantity in the interval i:

$$N_i^0 = \frac{n_i}{\rho v_i} = \frac{n}{\rho v_i}\omega_i \Delta r \qquad \text{* MERGEFORMAT (19)}$$

Because after molar quantity reduction in i interval by Δn_i the particle's number remains constant, we will get:

$$\frac{n_i}{v_i} = \frac{n_i - \Delta n_i}{v_i^*}$$
$$v_i\left(n_i - \Delta n_i\right) = v_i^* n_i \qquad \text{* MERGEFORMAT (20)}$$
$$v_i^* = v_i\left(1 - \frac{\Delta n_i}{n_i}\right)$$

v_i^* –volume of the particles after removing Δn_i moles of the substance. We will get:

$$v_i^* = \frac{4}{3}\pi\left(r^*\right)^3$$
$$v_i = \frac{4}{3}\pi r_i^3$$
$$\frac{4}{3}\pi\left(r^*\right)^3 = \frac{4}{3}\pi r_i^3\left(1 - \frac{\Delta n_i}{n_i}\right) \qquad \text{* MERGEFORMAT (21)}$$
$$r^* = r_i\left(1 - \frac{\Delta n_i}{n_i}\right)^{1/3}$$

Reduced particles goes to intervals j, $j+1$. Where:

$$j = \left\lfloor \frac{r^* - r_{\min}}{\Delta r} \right\rfloor \qquad \text{* MERGEFORMAT (22)}$$

Let's determine amount of substance distributed into these intervals. To do this we use two balance conditions: 1) balance of substance's amount; 2) balance of the total surface area of reduced particles:

$$N_j^* 4\pi r_j^2 + N_{j+1}^* 4\pi r_{j+1}^2 = N_i^0 4\pi r^{*2}$$

$$\frac{n_j^*}{v_j} r_j^2 + \frac{n_{j+1}^*}{v_{j+1}} r_{j+1}^2 = \frac{n_i}{v_i} r^{*2} \quad \text{* MERGEFORMAT (23)}$$

$$\frac{n_j^*}{r_j} + \frac{n_{j+1}^*}{r_{j+1}} = \frac{n_i}{r_i}\left(1 - \frac{\Delta n_i}{n_i}\right)^{2/3}$$

Balance of substance's amount:

$$n_j^* + n_{j+1}^* = n_i - \Delta n_i \quad \text{* MERGEFORMAT (24)}$$

From * MERGEFORMAT (23), * MERGEFORMAT (24) we get:

$$n_{j+1}^* = n_i - \Delta n_i - n_j^*$$

$$\frac{n_j^*}{r_j} + \frac{n_i - \Delta n_i - n_j^*}{r_{j+1}} = \frac{n_i}{r_i}\left(1 - \frac{\Delta n_i}{n_i}\right)^{2/3} \quad \text{* MERGEFORMAT (25)}$$

$$n_j^* = \frac{r_j}{\Delta r r_i}\left(r_{j+1} n_i \left(1 - \frac{\Delta n_i}{n_i}\right)^{2/3} - r_i\left(n_i - \Delta n_i\right)\right)$$

Finally:

$$n_j^* = n_j^* = \frac{r_j}{\Delta r r_i}\left(r_{j+1} n_i \left(1 - \frac{\Delta n_i}{n_i}\right)^{2/3} - r_i\left(n_i - \Delta n_i\right)\right) \quad \text{* MERGEFORMAT (26}$$

$$n_{j+1}^* = n_i - \Delta n_i - n_j^*$$

In case, when $\Delta n =\ n$, we obtain:

$$j = i - 1$$

$$n_i^{\uparrow} = \frac{r_{i-1}}{\Delta r}\left(n_i - \Delta n_i\right)^{2/3}\left(n_i^{1/3} - \left(n_i - \Delta n_i\right)^{1/3}\right) \text{* MERGEFORMAT (27)}$$

$$n_i^{\downarrow} = n_i - \Delta n_i - n_i^{\uparrow}$$

$n_i^{\uparrow}, n_i^{\downarrow}$ – amount of substance moved from interval i into interval $i-1$ amount of substance left in the interval i, respectively. Based on balance of substance's amount for interval i, we get:

$$n_i^* = n_i^{\downarrow} + n_{i+1}^{\uparrow}$$

$$\lambda_i = \frac{\omega_i}{r_i \sum_{j=0}^{m-1} \frac{\omega_j}{r_j}} \frac{\Delta n}{\Delta r}$$

* MERGEFORM

$$\omega_i^* = \omega_i - \frac{\lambda_i}{n} + \frac{1}{n} \frac{r_i}{\Delta r} \left(n\omega_{i+1} - \lambda_{i+1}\right)^{2/3} \left(\left(n\omega_{i+1}\right)^{1/3} - \left(n\omega_{i+1} - \lambda_{i+1}\right)^{1/3}\right) -$$

$$-\frac{1}{n} \frac{r_{i-1}}{\Delta r} \left(n\omega_i - \lambda_i\right)^{2/3} \left(\left(n\omega_i\right)^{1/3} - \left(n\omega_i - \lambda_i\right)^{1/3}\right)$$
